# Temporomandibular Disorders in 13‐ and 15‐Year‐Old Females: A Longitudinal Study

**DOI:** 10.1002/cre2.70257

**Published:** 2025-12-10

**Authors:** Christina Mejersjö, Eva‐Karin Bergström, Bengt Wenneberg, Anders Wänman

**Affiliations:** ^1^ Department of Orofacial Pain, Institute of Odontology University of Gothenburg Gothenburg Sweden; ^2^ Department of Cariology, Institute of Odontology University of Gothenburg Gothenburg Sweden; ^3^ Department of Preventive and Community Dentistry, Public Dental Service Region of Västra Götaland Gothenburg Sweden; ^4^ Institute of Odontology University of Gothenburg Gothenburg Sweden; ^5^ Department of Clinical Oral Physiology Umeå University Umeå Sweden

**Keywords:** adolescent girls, longitudinal study, signs, symptoms, temporomandibular disorders

## Abstract

**Objectives:**

Many adolescents, especially females, suffer from temporomandibular pain and dysfunction (TMD). We investigated the prevalence and progression of TMD symptoms in an early teen cohort in relation to oral parafunctions.

**Methods:**

Girls 13 years of age in 19 middle schools were invited to participate. A total of 630 girls were enrolled in a prospective study at age 13 and 507 girls were followed at the age of 15 years. The girls completed a short questionnaire at the start and at follow‐up. Clinical examination was performed at the start in 24% of the girls. All girls had regular check‐ups at the Community Dentistry Clinic.

**Results:**

Approximately one fourth of the 13‐year‐old girls reported TMD symptoms and headache once a week or more often. The frequencies increased during the follow‐up to one third of the girls at age 15 years. Headache was the most common complaint with daily headache occurring in 7.5% of the girls at 15 years. At the clinical examination at age 13, one or more TMD signs were noticed in 36% of the girls with pain‐related diagnoses in 32% (predominantly of muscle origin) and joint clicking in 4.5% of the girls. The need for some attention/care was estimated at 15%. There was a strong relationship between TMD symptoms and oral parafunctions.

**Conclusions:**

Approximately one third of 13‐ and 15‐year‐old girls reported TMD symptoms and had clinical signs. It is crucial to take heed to TMD symptoms in young teenagers.

## 1 Introduction

Many adolescents suffer from temporomandibular pain and dysfunction (Lövgren, Häggman‐Henrikson, et al. 2016; Christidis et al. 2019; Minervini et al. 2023).

Temporomandibular disorders (TMD) comprise several different types of pain and dysfunction symptoms that involve the jaw muscles, the temporomandibular joints (TMJ), and related structures (Suvinen et al. 2004; Okeson 2013). Orofacial pain, dysfunction of the TMJ, and decreased and/or painful mandibular movements are commonly reported complaints. Headache is often associated with the condition (Emshoff et al. 2017; Nilsson et al. 2013).

TMD symptoms have been noticed in 4%–44% of adolescents (Lövgren, Häggman‐Henrikson, et al. 2016; Christidis et al. 2019; Minervini et al. 2023; Da Silva et al. 2016; Ostansjo et al. 2017) with an increasing frequency with age. Girls are affected about twice as often as boys (Lövgren, Häggman‐Henrikson, et al. 2016; Christidis et al. 2019; Minervini et al. 2023; Okeson 2013). Children below 10 years of age rarely have TMD symptoms but the frequency increases during puberty, especially in females, with up to 20% being affected in their twenties. The background of the symptoms is complex and diverse and several risk factors for developing TMD symptoms have been identified (Nilsson et al. 2013; Da Silva et al. 2016; Ostansjo et al. 2017; Köhler et al. 2013; Marklund and Wänman 2010). Oral parafunctions were reported to be related to symptoms of the jaws and head (Ostansjo et al. 2017; Marklund and Wänman 2010; Motghare et al. 2015). Extended muscle tension is another background factor in the development of TMD symptoms. Psychological, comorbid, and socioeconomic factors have been identified to influence the risk of developing TMD symptoms (Suvinen et al. 2004; Fillingim et al. 2013; Magalhaes et al. 2014). TMD symptoms negatively influence the daily life of teenage girls, and among adults the symptoms were shown to decrease the quality of life and increase work absences (Mejersjö et al. 2024; Salinas Fredricson et al. 2022).

The treatment of TMD symptoms is generally effective and offers a favorable prognosis (Magnusson et al. 2005). Counseling has been reported to reduce significantly the symptoms in adult patients (Barros Pascoal et al. 2020). Muscle training is another effective treatment and was found to be beneficial in the management of TMD symptoms (Fisch et al. 2021; Asquini et al. 2022). Relaxation training reduces muscle tension both locally and generally, and it also reduces the frequency of headache (Meyer et al. 2016).

The aims of the study were to study the prevalence of TMD symptoms and signs among 13‐ and 15‐year‐old females, and to follow the progression of symptoms for 2 years in relation to certain risk factors.

## 2 Materials and Methods

### 2.1 The Study Population

Thirteen‐years‐old girls in the seventh grade of middle school in a western region of Sweden were invited to participate in a study of TMD symptoms and headache, as well as risk factors for the symptoms. Only girls were invited since the incidence of TMD symptoms and headache increases in girls from the age of 13 years of age and up (Lövgren, Häggman‐Henrikson, et al. 2016).

The administration of 21 schools in Västra Götaland, Sweden, were contacted, and 19 schools accepted to participate. The school level was compulsory and the schools were spread out geographically and socioeconomically in rural, suburban, and city areas. The girls were informed orally and in writing about the study, and written information was given to their parents that included a form to sign if they did not want their child to participate. Each girl was only identified by her name and class. No parent declined participation. The study was approved by the Ethics Committee of Gothenburg, reg. no. 457‐18.

**Figure 1:**
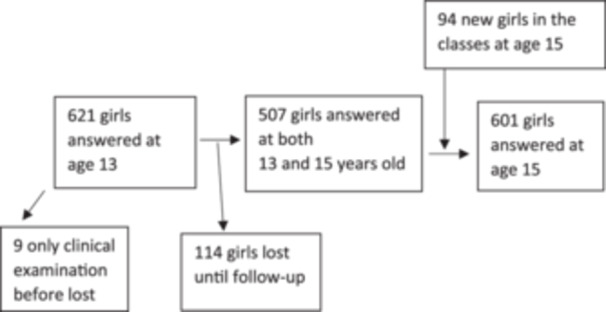
Flow chart of the 724 girls in the study (i.e., 9 + 621 + 94 = 724), 507 girls (81.6%) answered at both 13 and 15 years of age.

Six‐hundred thirty girls at aged 13 were included at the start of the seventh grade. Girls who could not speak Swedish were excluded. Inclusion was on going for September and October of 2019 and 2020. The study included all girls in the beginning of the seventh grade of the 19 schools studied. The plan was to follow the girls for 2–2.5 years. However, there were more girls than expected with some type of leave from the classes; also, there was a lot of relocation and change of schools during the follow‐up period. Some girls had stopped school either permanently or temporarily, and all together 114 girls were lost by the follow‐up at 15 years of age. When we returned to the 9th grade, all girls in the classes completed the questionnaire, including 94 girls who had not responded at 13 years of age. Some of these additional girls had been on some type of leave at 13 years of age but were able to respond at 15 years; and some others had moved to the classes during the time up to the 9th grade. In total, 507 girls participated at both 13 and 15 years of age. The 94 girls who only answered the second questionnaire at age 15 are reported separately (Figure 1).

### 2.2 The Study Methods

The girls answered a standardized questionnaire (Table 1) regarding symptoms of the jaws and head, and about oral habits (Saghafi and Mejersjö 2018). The questionnaire also included the three screening questions for TMD (3Q/TMD) (Lövgren, Visscher, et al. 2016):

Q1: “Do you have pain of the temple, face, jaw or jaw joint once a week or more often?”
Q2: “Do you have pain when you open your mouth or chew once a week or more often?”
Q3: “Does your jaw lock or become stuck once a week or more often?”

**Table 1:** The questionnaire regarding symptoms during the preceding 3 months.

1. Symptoms of TMD and frequency (never/rarely, 1–2 times/month, 1–2 times/week, 3–4 times/week, daily), tiredness of the jaws, joint sound, headache, pain of the face or jaws, difficulty to open wide, and locking of the jaw.
2. The three screening questions (3QTMD) (Lövgren, Visscher, et al. (2016)), symptoms, once/week or more often: yes/no.
3. Reported oral parafunctions and frequency (never/rarely, 1–2 times/month, 1–2 times/week, 3–4 times/week, daily), clenching or bruxing when awake, pressing the tongue to the teeth or palate, biting the lips, biting the nails, use of chewing gum.
4. General health on a five‐point scale (5=excellent to 1=bad).

The girls were also asked to estimate their self‐perceived general health status (Table 1).

The questionnaire was answered during an ordinary lesson in the class with their teacher and a dentist (co‐author C.M.) present. There was ample opportunity for the girls to ask questions if they had problems with the questionnaire.

At 13 years of age, 24% of the girls had a short clinical examination of their jaw function; (Mejersjö et al. 2018) this was performed by the same examiner for all the girls (co‐author B.W.) who was blinded to the answers in the questionnaire. The examination included maximum incisal opening (MIO), pain on opening, deviation on opening, TMJ clicking, TMJ tenderness on palpation and tenderness on extra oral palpation of the temporal and masseter muscles. A maximum mouth opening below 40 mm was regarded as impaired. The drop‐outs from this examination amounted to 8% and were girls who answered the questionnaire but did not appear at then clinical examination due to some type of leave. The clinical examination was not performed on the same day as the questionnaire was answered.

An estimated need for attention/care was calculated according to the clinical examination. The girls with obvious tenderness to palpation and/or more than one clinical sign of dysfunction were classified as having a need for attention.

After 2–2.5 years, at 15 years of age, a follow‐up was performed. All the girls in the classes, including the new girls, answered the questionnaire, in their class with a teacher present. No clinical examinations were performed for the girls at 15 years of age, because entry to some of the schools by outsiders was restricted during the Covid pandemic. All the girls in the study had regular check‐ups and treatment at the Community Dentistry Clinic for children and youth.

Statistical analyses of the questionnaires and the clinical examination were processed using the SPSS 20 (SPSS Inc., Chicago, IL) software. For comparison between symptoms, parafunctional habits and estimated general health, the chi‐square test and Student's *t*‐test were used. Statistically significant differences and correlations with a strength of *p* < 0.01 are presented, unless otherwise stated.

## 3 Results

### 3.1 Reported Symptoms

Symptoms reported from the temporomandibular system and the head at 13 and 15 years of age are presented in Table 2. There was a significant increase in all symptoms during the 2‐year period. The new girls in the classes at 15 years of age are reported separately. Headache was the most frequent symptom, affecting half of the girls once a week or more often at age 15.

**Table 2:** Reported symptom once/week or more often (including daily) and daily at 13 and 15 years of age for the 507 girls for the longitudinal part of the study, and *p* values for the change between ages. Also include are symptoms of the 94 girls who only answered at age 15. Data are percentage distributions.

Symptoms (%)	Longitudinal				*p*‐value	Only at 15 years	
	13 years		15 years				
	Once/w or more	(Daily)	Once/w or more	(Daily)		Once/w or more	(Daily)
Tired/stiff	8.4	(1.1)	17.1	(3.4)	0.001	24.7	(2.2)
TMJ clicking	14.3	(3.6)	16.7	(7.7)	0.001	24.8	(9.1)
Headache	48.4	(6.3)	51.1	(7.7)	0.001	62.7	(11.0)
Orofacial pain	6.9	(0.9)	11.4	(1.7)	0.001	11.1	(2.2)
Opening difficulties	5.2	(2.4)	5.8	(1.5)	0.004	12.4	(4.4)
Locking/catching	4.3	(1.0)	6.2	(2.2)	0.015	5.5	(2.2)

The three screening questions (3Q/TMD) showed a numerical increase during the period (Table 3). At 13 and 15 years of age, 29.6% and 32.3% of the girls, respectively, answered yes to one or more of the questions.

**Table 3:** Reported symptoms according to the screening questions (3Q/TMD) once/week or more often (yes or no) at 13 and 15 years of age for the 507 girls for the longitudinal part of the study. Also include are the 94 girls who only answered at age 15. Data are percentage distributions.

Screening question	Longitudinal (%)		Only at 15 years (%)
	13 years	15 years	
Q1: Facial pain	18.5	22.6	25.3
Q2: Pain at function	14.6	13.0	20.9
Q3: Locking or catching of the jaw	5.6	10.2	7.7

For Q1 and Q2 of the 3Q/TMD there was a strong correlation with all reported symptoms (*p* < 0.001), and Q3 also displayed this relationship (*p* < 0.001) except for headache.

### 3.2 Clinical Signs

One or more clinical signs were observed in 36% of the girls at age 13. The mean MIO was 49 mm (range 30–65 mm), with six girls (4%) measuring 30–38 mm. Muscle tenderness on palpation was found in 28% and joint tenderness in 4% of the girls. Joint tenderness was combined with muscle tenderness except for one girl with only joint tenderness. Pain‐related signs were found in 32% of the girls.

TMJ clicking was found in seven girls (4.5%). In three girls, clicking was combined with tenderness of either the TMJ or the jaw muscles or both, while in the other four girls there was no other clinical sign than the joint sound.

### 3.3 Reported Parafunctions

Oral parafunctions were frequently reported (Table 4). The reported symptoms significantly correlated with parafunctions of clenching, tongue pressure, and cheek biting (*p* < 0.001). Daily use of chewing gum correlated with the reported symptoms of feeling tired/stiff and feeling mouth opening difficulties (*p* = 0.003 and *p* = 0.015, respectively).

**Table 4:** Reported parafunctions once/week or more often (including daily) and daily at 13 and 15 years of age for the 507 girls for the longitudinal part of the study. Also include are parafunctions of the 94 girls who only answered at age 15. Data are percentage distributions.

Parafunction (%)	Longitudinal					Only at 15 years	
	13 years		15 years				
	Once/w or more	(Daily)	Once/w or more		(Daily)	Once/w or more	(Daily)
Clenching	38.1	(15.4)	42.5		(17.7)	51.7	(23.1)
Pressing the tongue	29.5	(11.7)	38.4		(16.3)	39.3	(23.6)
Biting the cheek	45.6	(17.3)	52.6		(22.7)	60.2	(28.4)
Nail biting	30.5	(12.5)	32.2		(12.3)	40.2	(13.8)
Use of chewing‐gum	84.0	(31.0)	77.6		(29.0)	80.4	(33.3)

The screening questions Q1 and Q2 (facial pain and pain on function) correlated with the parafunctions of clenching, tongue pressure, and cheek biting (*p* < 0.001). The use of chewing gum correlated with Q3 (jaw catching, *p* = 0.005), and nail biting correlated with Q2 and Q3 (*p* = 0.002).

### 3.4 Signs and Associated Symptoms

All girls with tenderness on palpation of the temporal muscle reported daily headache at age 13; and for those reporting headache 3–4 times/week, 67% had tenderness of the temporal muscle.

The need for dental attention due to pain and/or impaired function was estimated at 15% of the girls.

### 3.5 General Health

About one fourth of the girls regarded their self‐perceived general health as excellent and another fourth as bad. Impaired self‐perceived general health at 15 years of age correlated with reported clicking, locking and difficulty opening the mouth (*p* < 0.001), and with headache, orofacial pain and feeling tired/stiff (*p* = 0.002–0.019).

## 4 Discussion

Approximately 30% of the 15‐year‐old girls in the study reported TMD symptoms once a week or more often; the percentage was even higher for headache. The study confirms that the frequency of TMD symptoms is high in the early teens. The findings correspond to what was previously reported, (Christidis et al. 2019; Minervini et al. 2023) and the increase in symptoms during a 2‐year period is in accordance with other studies (Suvinen et al. 2004; Köhler et al. 2013). The association between headache and TMD is well documented, (Branco et al. 2013; Paolo et al. 2018) and this was confirmed in the present study by the close relation found between tenderness of the temporal muscle and headache.

The need for attention/care due to pain and/or impaired function was estimated at 15% of the girls. This estimated treatment need is in accordance with that found for non‐patients, albeit adults, with about 16%; (Al‐Jundi et al. 2008) however higher than that from a study reporting 8% (Köhler et al. 2013).

The number of symptoms reported by the 94 additional 15‐year‐old girls that had joined the classes until the second questionnaire was numerically more than the 15‐year‐olds followed longitudinally. One explanation could be that the group of new girls included those who were on sick‐leave during the first questionnaire, and of which some perhaps had a generally higher frequency of sick leave, while others had moved and changed classes, changes that could create tension.

Clinical signs were noticeable already at 13 years of age. The frequency of 32% of the girls having one or more clinical TMD signs corresponds to the 27%–40% found in other studies (Paduano et al. 2020; Restrepo et al. 2021). The most common diagnosis was pain‐related and of muscle origin, which complies findings by Restrepo et al. (2021) of 25.5% of subjects having myalgia at that age. In another study, a TMD diagnosis after clinical examination with DC/TMD (Schiffman et al. 2014) was found in 11.9% in an adolescent population (Graue et al. 2016).

The clinical examination method used (Mejersjö et al. 2018) is a short examination for TMD in general practice. Albeit short, it has shown to be in good agreement with DC/TMD examination. The short examination method made it possible to evaluate clinical signs in the limited time allowed by the schools for the study. Pain‐related diagnoses dominated and were mainly of muscular origin, whereas joint‐related diagnoses were few.

Our frequency of those with TMJ clicking was low compared with that at 10% (Da Silva et al. 2016) and 14.4% (Marpaung et al. 2019) found in other studies. This could be due to the short clinical examination method that allows detection of few mild joint sounds, and to the low age of the girls examined.

In the school situation with short and limited time available, the questionnaire used was considered suitable. The DC/TMD Axis II for children may have been suitable, (Rongo et al. 2022) however, it would not have covered all the questions of the study. The questionnaire used was a combination of parts of previously used questionnaires in dental studies (Saghafi and Mejersjö 2018), and parts from continuous studies of youth, (Hagquist 2008) with some parts validated.

During the study it was noticed that language comprehension was lacking to some degree and even ordinary Swedish words were unknown to some of the 13‐year‐old girls (e.g., “pain” and “temple”). In addition, there were several immigrant girls with less extensive knowledge of the language, even though we had excluded girls that did not know Swedish. Those circumstances point to the importance of the form of the questionnaire and how it relates to the age of the study population.

Headache was frequently reported by the girls in the study. It is sometimes difficult to distinguish between the diagnoses of tension headache and migraine, and muscle tension may be present in the background of both (Meyer et al. 2016). Headaches reported by the girls covered both diagnoses, and not all cases could be classified as tension headache associated with TMD.

Oral parafunctions correlated with reported symptoms of the jaws and head, which is in agreement with other studies (Ostansjo et al. 2017; Marklund and Wänman 2010; Motghare et al. 2015). This emphasizes the importance of the dental service to notice harmful habits, and to instruct and motivate the patients to work for a change. Other risk factors, which are more difficult to influence, include sociological and psychological factors (Suvinen et al. 2004; Fillingim et al. 2013). Nevertheless, the symptoms should be recognized, explained to the patient and appropriately dealt with.

Some of the TMD symptoms correlated with the girls' own report on their general health status which indicates the importance of TMD symptoms for anxiety and well‐being, and which is in accordance with what was found in other studies (Mejersjö et al. 2024; Marpaung et al. 2024).

The study was partly performed under the COVID pandemic. In Sweden, however, schools continued as usual with teaching, gymnastics and food, and were not closed; this minimized the impact of COVID on the girls' life. Entry to schools was only restricted to external personnel.

The girls participating both at 13 and 15 years of age are considered representative of young teenage girls in the Region of Västra Götaland, Sweden. The drop outs were many, as expected in a prospective longitudinal study; however, the level was regarded acceptable at about 18%. A strength of the study is that the group of girls who were followed for 2–2.5 years is fairly large, and that the questionnaire was combined with a clinical examination in 24% of the girls.

## 5 Conclusion

Girls in their lower teens present a great deal of TMD symptoms and signs, with an increasing prevalence during a 2‐year period, and with an obvious need for treatment in this age span. There is a strong relationship between reported symptoms and parafunctions.

## Author Contributions

Christina Mejersjö initiated the study, performed the study, and wrote the manuscript. Eva‐Karin Bergström made the study possible and commented on the manuscript. Bengt Wenneberg performed the clinical examination and commented on the manuscript. Anders Wänman initiated the study and wrote the manuscript.

## Ethics Statement

The study was approved by the Ethics Committee of Gothenburg, reg. no. 457‐18.

## Conflicts of Interest

The authors declare no conflicts of interest.

## Data Availability

The research data associated with the paper are available from the authors Professor Anders Wänman anders.wanman@umu.se and Docent Christina Mejersjö christina.mejersjo@gu.se and the data in the SPSS‐files are available from the authors on request.

## References

[cre270257-bib-0001] Al‐Jundi, M. A. , M. T. John , J. M. Setz , A. Szentpétery , and O. Kuss . 2008. “Meta‐Analysis of Treatment Need for Temporomandibular Disorders in Adult Nonpatients.” Journal of Orofacial Pain 22, no. 2: 97–107. 18548838

[cre270257-bib-0002] Asquini, G. , L. Pitance , A. Michelotti , and D. Falla . 2022. “Effectiveness of Manual Therapy Applied to Craniomandibular Structures in Temporomandibular Disorders: A Systematic Review.” Journal of Oral Rehabilitation 49, no. 4: 442–455. 34931336 10.1111/joor.13299

[cre270257-bib-0003] Barros Pascoal, A. L. , R. F. Carvalho Porto de Freitas , L. F. Grangeiro da Silva , et al. 2020. “Effectiveness of Counseling on Chronic Pain Management in Patients With Temporomandibular Disorders.” Journal of Oral & Facial Pain and Headache 34, no. 1: 77–82. 30978270 10.11607/ofph.2163

[cre270257-bib-0004] Christidis, N. , E. Lindström Ndanshau , A. Sandberg , and G. Tsilingaridis . 2019. “Prevalence and Treatment Strategies Regarding Temporomandibular Disorders in Children and Adolescents—A Systematic Review.” Journal of Oral Rehabilitation 46, no. 3: 291–301. 30586192 10.1111/joor.12759

[cre270257-bib-0005] Emshoff, R. , F. Bertram , D. Schnabl , and I. Emshoff . 2017. “Association Between Chronic Tension‐Type Headache Coexistent With Chronic Temporomandibular Disorder Pain and Limitations in Physical and Emotional Functioning: A Case‐Control Study.” Journal of Oral & Facial Pain and Headache 31: 55–60. 28118421 10.11607/ofph.1654

[cre270257-bib-0006] Fillingim, R. B. , R. Ohrbach , J. D. Greenspan , et al. 2013. “Psychological Factors Associated With Development of TMD: The OPPERA Prospective Cohort Study.” Journal of Pain 14, no. S12: T75–T90. 24275225 10.1016/j.jpain.2013.06.009 PMC3855656

[cre270257-bib-0007] Fisch, G. , A. Finke , J. Ragonese , L. Dugas , and M. Wrzosek . 2021. “Outcomes of Physical Therapy in Patients With Temporomandibular Disorder: A Retrospective Review.” British Journal of Oral and Maxillofacial Surgery 59, no. 2: 145–150. 33280944 10.1016/j.bjoms.2020.08.068

[cre270257-bib-0008] Graue, A. M. , A. Jokstad , J. Assmus , and M. S. Skeie . 2016. “Prevalence Among Adolescents in Bergen, Western Norway, of Temporomandibular Disorders According to the DC/TMD Criteria and Examination Protocol.” Acta Odontologica Scandinavica 74: 449–455. 27251463 10.1080/00016357.2016.1191086

[cre270257-bib-0009] Hagquist, C. 2008. “Psychometric Properties of the Psychosomatic Problems Scale: A Rasch Analysis on Adolescent Data.” Social Indicators Research 86, no. 3: 511–523.

[cre270257-bib-0010] Köhler, A. A. , A. Hugoson , and T. Magnusson . 2013. “Clinical Signs Indicative of Temporomandibular Disorders in Adults: Time Trends and Associated Factors.” Swedish Dental Journal 37, no. 1: 1–11. 23721032

[cre270257-bib-0011] Lövgren, A. , B. Häggman‐Henrikson , C. M. Vischer , et al. 2016. “Temporomandibular Pain and Jaw Dysfunction at Different Ages Covering the Lifespan—A Population‐Based Study.” European Journal of Pain 20: 532–540. 26311138 10.1002/ejp.755

[cre270257-bib-0012] Lövgren, A. , C. M. Visscher , B. Häggman‐Henrikson , F. Lobbezoo , S. Marklund , and A. Wänman . 2016. “Validity of Three Screening Questions (3Q/TMD) in Relation to the DC/TMD.” Journal of Oral Rehabilitation 43, no. 10: 729–736. 27573533 10.1111/joor.12428

[cre270257-bib-0013] Magalhaes, B. , S. DeSousa , V. DeMello , et al. 2014. “Risk Factors for Temporomandibular Disorder: Binary Logistic Regression Analysis.” Medicina Oral Patología Oral y Cirugia Bucal 19, no. 3: e232–e236. 24316706 10.4317/medoral.19434 PMC4048110

[cre270257-bib-0014] Magnusson, T. , I. Egermark , and G. E. Carlsson . 2005. “A Prospective Investigation Over Two Decades on Signs and Symptoms of Temporomandibular Disorders and Associated Variables. A Final Summary.” Acta Odontologica Scandinavica 63: 99–109. 16134549 10.1080/00016350510019739

[cre270257-bib-0015] Marklund, S. , and A. Wänman . 2010. “Risk Factors Associated With Incidence and Persistence of Signs and Symptoms of Temporomandibular Disorders.” Acta Odontologica Scandinavica 68: 289–299. 20528485 10.3109/00016357.2010.494621

[cre270257-bib-0016] Marpaung, C. , M. K. A. van Selms , and F. Lobbezoo . 2019. “Temporomandibular Joint Anterior Disc Displacement With Reduction in a Young Population: Prevalence and Risk Indicators.” International Journal of Paediatric Dentistry 29, no. 1: 66–73. 30218477 10.1111/ipd.12426

[cre270257-bib-0017] Marpaung, C. , A. U. Yap , I. Hanin , and A. Fitryanur . 2024. “Psychological Distress and Well‐Being: Their Association With Temporomandibular Disorder Symptoms.” CRANIO 42, no. 3: 285–291. 34432614 10.1080/08869634.2021.1971449

[cre270257-bib-0018] Mejersjö, C. , E. K. Bergström , C. Hagquist , and A. Wänman . 2024. “Impact of Temporomandibular Disorder Symptoms Among 15‐Year‐Old Girls.” Acta Odontologica Scandinavica 83, no. 8: 441–445. 10.2340/aos.v83.41113. 39177399 PMC11407104

[cre270257-bib-0019] Mejersjö, C. , O. Bertilsson , and K. Bäck . 2018. “Short Clinical Examination for Temporomandibular Symptoms in General Practice.” Acta Odontologica Scandinavica 76, no. 7: 183–187. 29140141 10.1080/00016357.2017.1401657

[cre270257-bib-0020] Meyer, B. , A. Keller , H. G. Wöhlbier , C. H. Overath , B. Müller , and P. Kropp . 2016. “Progressive Muscle Relaxation Reduces Migraine Frequency and Normalizes Amplitudes of Contingent Negative Variation (CNV).” Journal of Headache and Pain 17: 37. 27090417 10.1186/s10194-016-0630-0 PMC4835398

[cre270257-bib-0021] Minervini, G. , R. Franco , M. M. Marrapodi , L. Fiorillo , G. Cervino , and M. Cicciù . 2023. “Prevalence of Temporomandibular Disorders in Children and Adolescents Evaluated With Diagnostic Criteria for Temporomandibular Disorders: A Systematic Review With Meta‐Analysis.” Journal of Oral Rehabilitation 50: 522–530. 10.1111/joor.13446. 36912441

[cre270257-bib-0022] Motghare, V. , J. Kumar , S. Kamate , et al. 2015. “Association Between Harmful Oral Habits and Sign and Symptoms of Temporomandibular Joint Disorders Among Adolescents.” Journal of Clinical and Diagnostic Research 9, no. 8: ZC45–ZC48. 26436046 10.7860/JCDR/2015/12133.6338 PMC4576640

[cre270257-bib-0023] Nilsson, I. M. , T. List , and M. Drangsholt . 2013. “Headache and Co‐Morbid Pains Associated With TMD Pain in Adolescents.” Journal of Dental Research 92: 802–807. 23813050 10.1177/0022034513496255

[cre270257-bib-0024] Okeson, J. P. 2013. “Signs and Symptoms of Temporomandibular Disorders.” In Management of Temporomandibular Disorders and Occlusion, edited by J. P. Okeson , 7th ed., 129–169. Elsevier Inc.

[cre270257-bib-0025] Ostansjo, V. , K. Moen , T. Storesund , et al. 2017. “Prevalence of Painful Temporomandibular Disorders and Correlation to Lifestyle Factors Among Adolescents in Norway.” Pain Research and Management 2017: 2164825. 28638246 10.1155/2017/2164825 PMC5468573

[cre270257-bib-0026] P. Branco, L. , T. O. Santis , T. A. Alfaya , C. H. L. Godoy , Y. D. Fragoso , and S. K. Bussadori . 2013. “Association Between Headache and Temporomandibular Joint Disorders in Children and Adolescents.” Journal of Oral Science 55, no. 1: 39–43. 23485599 10.2334/josnusd.55.39

[cre270257-bib-0027] Paduano, S. , R. Bucci , R. Rongo , R. Silva , and A. Michelotti . 2020. “Prevalence of Temporomandibular Disorders and Oral Parafunctions in Adolescents From Public Schools in Southern Italy.” CRANIO 38, no. 6: 370–375. 30547719 10.1080/08869634.2018.1556893

[cre270257-bib-0028] Paolo, C. , A. D'Urso , P. Papi , et al. 2018. “Temporomandibular Disorders and Headache: A Retrospective Analysis of 1198 Patients.” PLoS One 13, no. 2: e0192254. 28420942 10.1155/2017/3203027 PMC5379086

[cre270257-bib-0029] Restrepo, C. , A. M. Ortiz , A. C. Henao , and R. Manrique . 2021. “Association Between Psychological Factors and Temporomandibular Disorders in Adolescents of Rural and Urban Zones.” BMC Oral Health 21, Article number: 140. 33743662 10.1186/s12903-021-01485-4 PMC7981971

[cre270257-bib-0030] Rongo, R. , E. Ekberg , I. M. Nilsson , et al. 2022. “Diagnostic Criteria for Temporomandibular Disorders in Children and Adolescents: An International Delphi Study‐Part 2‐Development of Axis II.” Journal of Oral Rehabilitation 49, no. 5: 541–552. 34951729 10.1111/joor.13301

[cre270257-bib-0031] Saghafi, E. , and C. Mejersjö . 2018. “A Method for Preventive Intervention Regarding Temporomandibular Pain and Dysfunction.” Acta Odontologica Scandinavica 76, no. 7: 482–487. 29448878 10.1080/00016357.2018.1439529

[cre270257-bib-0032] Salinas Fredricson, A. , C. Krüger Weiner , J. Adami , et al. 2022. “Sick Leave and Disability Pension in a Cohort of TMD‐Patients—The Swedish National Registry Studies for Surgically Treated TMD.” BMC Public Health 22: 916. 10.1186/s12889-022-13239-z. 35534826 PMC9082829

[cre270257-bib-0033] Schiffman, E. , R. Ohrbach , E. Truelove , et al. 2014. “Diagnostic Criteria for Temporomandibular Disorders (DC/TMD) for Clinical and Research Applications: Recommendations of the International RDC/TMD Consortium Network and Orofacial Pain Special Interest Group.” Journal of Oral & Facial Pain and Headache 28, no. 1: 6–27. 24482784 10.11607/jop.1151 PMC4478082

[cre270257-bib-0034] Da Silva, C. G. , C. Pachêco‐Pereira , A. L. Porporatti , et al. 2016. “Prevalence of Clinical Signs of Intra‐Articular Temporomandibular Disorders in Children and Adolescents.” Journal of the American Dental Association 147: 10–18.e8. 26552334 10.1016/j.adaj.2015.07.017

[cre270257-bib-0035] Suvinen, T. I. , M. Nyström , M. Evälahti , E. Kleemola‐Kujala , A. Waltimo , and M. Könönen . 2004. “An 8‐Year Follow‐Up Study of Temporomandibular Disorder and Psychosomatic Symptoms From Adolescence to Young Adulthood.” Journal of Orofacial Pain 18: 126–130. 15250432

